# Nutritional and performance viability of cactus *Opuntia*-based diets with different concentrate levels for Girolando lactating dairy cows

**DOI:** 10.5713/ajas.18.0916

**Published:** 2019-05-27

**Authors:** Jonas Gomes Inácio, Maria Gabriela da Conceição, Djalma Cordeiro dos Santos, Júlio César Vieira de Oliveira, Juana Catarina Cariri Chagas, Gláucia Sabrine de Oliveira Moraes, Evannielly Thuanny dos Santos Silva, Marcelo de Andrade Ferreira

**Affiliations:** 1 Federal Rural University of Pernambuco, Department of Animal Science, 52171-900 Recife, PE, Brazil; 2 Agronomic Institute of Pernambuco, 56500-000 Arcoverde, PE, Brazil; 3 Department of Agricultural Research of Northern Sweden, Swedish University of Agricultural Sciences, 90183 Umeå, Sweden

**Keywords:** *Cactacea*, Dairy Cattle, Feeding Efficiency, Semiarid

## Abstract

**Objective:**

The aim of this research was to evaluate the effect of different concentrate levels in diets based on cactus *Opuntia Stricta* (Haw.) Haw cladodes on the performance of lactating Girolando cows.

**Methods:**

The experiment involved 10 Girolando multiparous dairy cows at 512.6 kg of body weight (BW) and producing 13.2 kg milk/d, allocated into two 5×5 Latin squares. The experimental treatments consisted of control diet composed by cactus *Nopalea cochenillifera*. Salm-Dyck. cladodes (*Nopalea*), forage sorghum silage and concentrate at 20% on dry matter (DM) basis, and four concentrate levels diets (20%, 24%, 28%, and 32%) plus cactus *Opuntia stricta* (Haw.) Haw. cladodes (*Opuntia*) and forage sorghum silage.

**Results:**

Regarding cows fed control diet, the nutrients intake were greater than for cows fed with cactus *Opuntia* and concentrate. Regarding concentrate levels, intakes of DM, organic matter (OM), crude protein (CP), non-fiber carbohydrates (NFC), and total digestible nutrients of cows increased linearly. Organic matter, CP, and NDF digestibilities were similar in between to control diet and cactus *Opuntia*-based diets. The digestibility of NFC increased linearly when the concentrate was inserted. The N balance was the same for control diet and cactus *Opuntia*-based diets, irrespective the concentrate levels.

**Conclusion:**

For cows producing 14 kg/d with 3.5% of fat, it is recommended 32% of concentrate to be included in cactus *Opuntia*-based diets, and the increase in concentrate level promotes a linear increase in milk yield.

## INTRODUCTION

Dairy cattle production is one of the few economically possible activities in the semiarid regions of Brazilian northeast, in which feeding the heard is characterized by the usage of cultivated forage and native vegetation [1]. The prevailing production system is characterized by smallholders system with an average of the productive area of 37 ha [2]. Nevertheless, the reduced size of the property has limited the production of forage, thus causing difficulties in the increase of milk productivity per area.

Forage cactus due to its high production of green matter per unit of area, therefore becoming fundamental for the increase of the efficiency of the land’s productivity. The cactus can be used when added to a source of fiber [3] and as a source of energy [4] when its nutritional deficiencies are corrected [1].

The superiority of cactus *Nopalea cochenillifera*. Salm-Dyck. cladodes (*Nopalea*) in relation to other genotypes mainly when compared to the cactus *Opuntia stricta* (Haw.) Haw cladodes (*Opuntia*) was already observed [5]. However, from the agronomic point of view, the cactus *Opuntia* has presented less demand of nutrients, it has been more tolerant to the conditions of hydric stress and it has also presented a higher production of dry matter per unit area (37 t of DM/ha/2 yr) than cactus *Nopalea* (21 t of DM/ha/2 yr) [6], sparking more interest in its insertion in the feeding of dairy cows in the semiarid.

A problem observed by Rocha Filho [7] for the inclusion of cactus *Opuntia* in the diet of lactating cows was the decreased consumption of nutrients and of milk production, associated to low acceptability when compared to diets with cactus *Nopalea*. As an alternative to solve this problem it would be increasing the proportion of concentrate in diets with cactus *Opuntia* to compensate the lessened consumption. Thus, it was hypothesized the existence of concentrate level associated to the cactus *Opuntia* which maximizes performance in lactating dairy cows.

The aim of this research was to evaluate the effect of concentrate levels in diets based on cactus *Opuntia Stricta* (Haw.) Haw cladodes over the performance of lactating Girolando cows.

## MATERIALS AND METHODS

The study was approved by the Ethics committee of Federal Rural University of Pernambuco (License n° 069/2016) and was conducted at Experimental Station of the Instituto Agronômico de Pernambuco (IPA), located at Arcorverde, Pernambuco, BR, presenting a semiarid climate “Bsh”.

The experiment involved 10 Girolando multiparous dairy cows at 512.6±53.66 kg of body weight (BW), 14 weeks in milking and producing 13.2±1.94 kg milk/d, allocated into two 5×5 simultaneous Latin squares. The trial lasted for 126 days, with four consecutive 21-day periods divided into 14-day adaptation and seven-day sampling periods.

The individual BW was measured at the beginning and at the end of each experimental period after milking. The cows were housed in individual pens, with approximately 24 m^2^, with individual feed bunks and unrestricted access to water. The experimental treatments consisted of control diet composed by cactus *Nopalea cochenillifera*. Salm-Dyck. cladodes (*Nopalea*), forage sorghum silage and concentrate at 20% on dry matter (DM) basis [7], and four concentrate levels diets (20%, 24%, 28%, and 32%) plus cactus *Opuntia stricta* (Haw.) Haw. cladodes (*Opuntia*) and forage sorghum silage. The diets were formulated to be isonitrogenous (13.3% crude protein; CP) and meet dairy cows’ nutrients requirements producing 14 kg of milk with 3.5% of fat [8], considering the ingredients composition (Table 1).

Feed was supplied *ad libitum* as total mixed ration, twice a day at 8:00 and 16:00, allowing 5% to 10% in orts (DM basis). Tables 1 and 2 present the diets feeds chemical composition, diets composition percentages and diets chemical composition. The voluntary intake was evaluated from the 15th to 21th day. In this sense, the amounts of supplied diet and orts were taken into account. The diet ingredients and orts samples were pooled per animal and experimental period and stored in plastic bags at −20°C. At the end of the experiment, the samples were oven-dried at 60°C for 72 hours and ground to pass through a 2 mm mesh for in situ ruminal incubation and through a 1 mm screen for further chemical analyses.

For estimated apparent digestibility and total digestible nutrient (TDN) concentration, the spot fecal samples were collected directly from the animals’ rectums in between the 16th and 20th days of each experimental period [9], and the samples were pooled per animal, and experimental period and stored at −20°C for chemical analyzes. The total fecal excretion was estimated using the indigestible neutral detergent fiber (iNDF) as an internal marker, and the feces, feed and orts iNDF content were obtained after 288 hours of ruminal incubation time [10]. The diets TDN content and its conversion in lactation net energy were estimated according [11,8].

After sample processing to pass through a 1 mm screen sieve, we evaluated for DM (method INCT-CA G-003/1), organic matter (OM, method INCT-CA M-001/1), CP (method INCT-CA N-001/1), ether extract (EE, method INCT-CA G-005/1), neutral detergent fiber (NDF) corrected for ash and protein (NDFap, methods INCT-CA F-002/1, INCT-CA M- 002/1, and INCT-CA N-004/1), and neutral detergent insoluble protein (NDIP), method INCT-CA N-004/1), according to the standard techniques of the Brazilian National Institute of Science and Technology in Animal Science [12]. The quantification of non-fiber carbohydrates (NFC) was performed according to Detmann and Valadares Filho [13] as follows: NFC = OM–([% CP–% CP of urea+% urea]+% NDFap+% EE+% MM).

Blood samples were taken on the 21th day of each sampling period, four hours after the morning feeding, from the coccygeal vein of each animal. Samples were immediately centrifuged at 2,100× g/min for a period of 15 min, and the remaining plasma or blood serum was maintained at −20°C for further analysis of urea concentration.

Simultaneously to blood sampling, spot urine samples were collected of each cow [14]. The urine was filtered through gauze and an aliquot of 10 mL was diluted immediately in 40 mL of H_2_SO_3_ (0.036 N). The samples were stored at −20°C for further nitrogen, urea, allantoin (AL), uric acid (UA), and creatinine analysis.

To analyze AL in milk and urine, we used the colorimetric method as described by Chen and Gomes [15]. The urea concentration in urine was analyzed via the enzymatic-colorimetric system of the urease method, using commercial kits (Labtest Diagnóstica S.A. Lagoa Santa, MG, Brazil). The UA concentration in urine was analyzed via the enzymatic Trinder method, using commercial kits (Labtest Diagnóstica S.A., Brazil). The creatinine concentration in urine was analyzed using “end point” markers with picrate and acidification, using commercial kits (Labtest Diagnóstica S.A., Brazil).

Daily total urinary volume was estimated through the relation of daily urinary excretion of creatinine, using the observed values of creatinine concentration in urine as described by Valadares Filho and Valadares [16]. The daily urinary excretion of creatinine was based on 24.05 mg/kg of BW of creatinine [14]. Microbial protein synthesis was estimated according Chen and Gomes [15], considering a recovery of absorbed purines of 0.85 and an endogenous contribution to the excretion of purines as recommended by González-Ronquillo et al [17].

The nitrogen balance (NB) was obtained through the differences between total nitrogen intake (N intake) and feces (N fecal), milk (N milk), and urine (N urine) total nitrogen. The N milk was quantified using milk total protein (MTP/6.38), and the milk urea nitrogen (MUN) was estimated using the equation N urine (g/d) = 12.54×N milk (mg/dL).

The cows were milked twice a day (6:00 and 15:00), and the milk yield (MY) was registered from the 15th to the 21th day of each experimental period. Milk samples were collected on days 18th and 19th of both milking periods, after last collection, and composed samples were made for each cow. A milk aliquot of 50 mL was conditioned in plastic bottles with preservative (Bronopol, D & F Control Systems, Inc., New York, NY, USA), maintained between 2°C and 6°C, and sent to the PROGENE Laboratory for evaluation of lactose, fat, protein, total solids, casein, and urea, following the methods of ISO [18]. Another 10 mL aliquot of milk was deproteinated with 5 mL of trichloroacetic acid (25%), filtered, and stored at −15°C for further AL analysis. The 3.5% fat-corrected milk yield (FCMY) was estimated as the equation FCMY (3.5%) = ([0.432+0.1625×% milk fat]×MY kg/d) [19].

The data were submitted for analysis of variance and regression using the MIXED procedure of the statistical program SAS (version 9.4, SAS Institute Inc., Cary, NC, USA), adopting 5% as significance level for the type I error, according to the following model:

$${\text{Y}}_{\text{ijkl}}=\mu +{\text{T}}_{\text{i}}+{\text{S}}_{\text{j}}+{\left(\text{P}/\text{S}\right)}_{\text{jk}}+{\left(\text{A}/\text{S}\right)}_{\text{jl}}+{ɛ}_{\text{ijkl}}$$

Where: Y_ijkl_ = observation ijkl; μ = over mean; T_i_ = fixed effect of treatment i; S_j_ = random effect of square j; (P/S)_jk_ = random effect of period k within the square j; (A/S)_il_ = random effect of animal l within the square j; ɛ_ijkl_ = random residual error associated with each observation, assuming the NID (0; σ2).

Dunnett test was used to compare each treatment group mean (concentrate levels), with the average of control diet. Comparisons between concentrate levels in the diets were conducted by the decomposition of sum of squares in orthogonal linear contrasts, and quadratic effects at 5% probability, with subsequent adjustments of the regression equations.

## RESULTS AND DISCUSSION

The control diet provided 14 kg of milk with 3.5% of fat as expected. The cows fed with the control diet presented lower intake for most nutrients (DM, OM, CP, NFC, and TDN), except for NDF, than those which received diets with cactus *Opuntia*, regardless of the concentrate levels. The concentrate inclusion of cactus *Opuntia*-based diets allowed for a linear increase in the intakes of DM, OM, CP, NFC, and TDN. Nevertheless, the NDF intake remained unaltered (Table 3).

The digestibility of DM was greater for the control diet than for the diet with cactus *Opuntia* and 20% of concentrate. The increase of concentrate in cactus *Opuntia*-based diets did not alter the digestibility of nutrients (Table 3).

It was observed a similarity of data collected in the present study with those obtained by Rocha Filho [7] which served as a base for the control diet; the intake of DM (14.96 and 12.11 kg/d) and the MY (14.0 and 13.0 kg/d) for cactus *Nopalea* and *Opuntia*, respectively.

Regarding the proportion of leftovers observed (10.46%) the levels of the different ingredients remained unaltered. Based on this assumption and considering that the cows did not selected the feeds, it was clear the inhibitory effect of DM intake for the genotype of cactus *Opuntia*. [5,7] also observed this effect. Nevertheless, in data presented by Monteiro et al [20], this effect was not observed. The difference lies in the proportion of concentrate which was significantly higher (30%), thus there was a lesser proportion of cactus (39%).

Even though it was not quantified other causes that could be aroused would be the interference of the malic acid. The increase in the diet malic acid concentration diminishes the palatability intake of DM, which can have potentially negative effects on the animal performance [21]. Also, the cactus Opuntia presents more organic acids content than other genotypes implying in acidic smell after chopped [22], a fact that may negatively interfere in the feed intake.

The fixation of CO_2_ in the plants Crassulacean acid metabolism (CAM) only occurs at night, in the dark, when the stomata are open. In this moment, the fixation of CO_2_ in phosphoenolpyruvate to form oxaloacetate. This last substance is rapidly transformed in malate and stored all night long in the vacuoles in the form of malic acid. When the day breaks the stomata close themselves and the malic acid is removed from the vacuole, transported to the chloroplast of the cell and decarboxylated, thus, producing pyruvate and CO_2_. The fixed CO_2_ is transferred to ribulose 1.5-bisphosphate of the Calvin’s cycle. The pyruvate produced can be converted in sugar and starch.

Therefore, it is possible to infer that in the CAM plants the formation of malic acid occurs at night and its consumption during the day. This causes a change in the taste of the plant during the day because at night an acid taste is observed. During the day the plant becomes sweeter. It is important to highlight that the first feed was offered at 8 h. This fact seems to be more evident in the cactus *Opuntia*. Silva et al [22] measured both the pH of the cactus *Nopalea* and *Opuntia*, harvested in the morning and a significantly lesser pH was observed in the cactus *Opuntia* (4.59) in relation to cactus *Nopalea* (5.01).

Despite the DM intake of *Opuntia* plus concentrate diets had been lower than DM intake of control diet, MY was guaranteed, and the BW gain was 22, 36, 40, and 70 g/d for diets with 20%, 24%, 28%, and 32% of concentrate inclusion, respectively. Also, the balance of nutrients presented explains the similarity in the MY observed between the control diet and the diet with cactus *Opuntia* and 32% of concentrate. However, the highest DM intake for control diet can be beneficial after lactating peak for recover the body reserves and preparing the body condition for the next calving, considering that cows fed control diet gained 200 g/d during the experiment.

Regarding NDF there was a compensation, that is to say with the increase in the proportion of the concentrate there was a decrease in the levels of fiber in the diets (Table 2) which probably was one of the factors that stimulated the rise in the consumption of DM which is normally justified by a higher concentration of fast-digesting ruminal carbohydrates (i.e. NFC) [23], as observed by Inácio et al [24] who tested different concentrate levels for heifers fed sugarcane bagasse as an exclusive roughage source. On the other hand, Chung et al [25] relate the increase in nutrients intake to the higher physical density of the concentrate by the decrease in the size of the particles in relation to the roughage. The main effects are the augmentation of the passage rate of the digesta through the gastrointestinal tract, making the increase in the consumption possible.

It was not found any difference between the control diet and the different levels of concentrate in the diets based on cactus *Opuntia* for the different sanguine parameters. The concentrations of blood urea nitrogen (BUN), urea and glucose were not altered with the inclusion of concentrate in the diets based on cactus *Opuntia*. On the other hand, the concentrations of non-esterified fatty acids (NEFA) and β-hydroxybutyrate (BHBA) decreased linearly (Table 4).

The NEFA and BHBA are important metabolic parameters to measure the nutritional status and of the adaptation to negative energetic balance of dairy cows during the body tissue mobilization [26]. The levels of NEFA observed (Table 4) were lower than the levels which are considered as normal (0.60 mmol/L) by Enjalbert et al [27], however, above this value it indicates an augmentation of the risk of occurrence of metabolic diseases as abomasum displacement, clinic ketosis, metritis and placenta retention.

The BHBA is important in dairy cattle as an indicator of subclinic ketosis, caused by the mobilization of body fat to meet the energetic deficit [28]. The concentration of BHBA observed in the present study was below the level considered as an indicator of subclinic ketosis less than 10 mg/dL or 0.97 mmol/L by Enjalbert et al [27], which indicates other metabolic conditions indicating there was not fat mobilization. Regarding the BUN of the control diet (22.9 mg/dL) and of the diets with cactus *Opuntia* (22.3, 23.2, 21.4, and 23.7 mg/dL) (Table 4), they are above the 19 mg/dL limit, which indicates loss of dietetic nitrogen in the cows [29] which shows the inefficiency of the usage of dietetic protein by these animals.

It was not observed difference between the control diet and the different levels of concentrate in the diets with cactus *Opuntia* for urinary volume, urea urine, BUN, MUN, microbial nitrogen, microbial CP and efficiency of microbial protein synthesis which also remained unaltered due to the inclusion of concentrate in the diets with cactus *Opuntia* (Table 5).

The absence of variation in estimated values of the synthesis and synthesis efficiency of the microbial CP (Table 5) with the offer of diets containing cactus cladodes can be explained by the amount of diet carbohydrates (Table 2), which were sufficient for supplying the necessary energy for the fermentation of fiber and the microbial synthesis. The N urea in milk (12.53, 13.39, 12.08, 11.54, and 12.16 mg/dL), are within the variation of 12 to 17 mg/dL, which, according to Abrahamse et al [30], values within this variation would indicate adequate balance of degraded protein and fermented energy in the rumen.

The NB observed in cows fed with the control diet was similar to those which received the diet based on cactus *Opuntia*, regardless of concentrate level. The N excretion in the milk was greater for control diet in relation to those with cactus *Opuntia* with 20% and 24% of concentrate. The N intake and the N fecal in milk increased linearly with the inclusion of concentrate in the diets with cactus *Opuntia*, thus not affecting the N fecal and N urine (Table 6).

In spite of the change in the N intake, regarding the NB, the similarity of the results observed not only in animals receiving the control diet but also those supplemented with concentrate and even within the levels, they can be explained by a higher excretion via milk or by the amount of milk produced as well as by the higher proportion of protein (Table 6).

The FCMY of cows fed with 32% of concentrate in the diet based on cactus *Opuntia* was similar to those which received the control diet (Table 7). The FCMY increased linearly with the insertion of concentrate in the diets based on cactus *Opuntia* (Table 7). The levels of fat, protein, total solids (TS) were higher for the control diet when compared to diets based on cactus *Opuntia*. When concentrate was included in the diets with cactus *Opuntia* it was not affected the content of fat, protein, lactases, TS, casein, urea nitrogen and feed efficiency (Table 7). The efficiency of concentrate usage in the control diet was less than in the diet with 20% of concentrate and higher than the levels of 24%, 28%, and 32% of concentrate (Table 7). As the levels of concentrate in the diets were increased, the efficiency of usage of the concentrate diminished linearly (Table 7).

The concentrate inclusion caused an increase in the nutrient’s intake, thus in the MY, without altering the level of fat. Only the diet with the highest level of concentrate (32%) provided a production of milk identical to that verified for control diet (Table 7), which can be explained by the similarity in the nutrient’s intake, notably TDN (Table 3).

The higher amount of protein (3.5 g/100 g) of milk for control when compared to the others experimental diets (3.25, 3.26, 3.27, 3.32 g/100 g), is related to a higher quantity of NFC of the control diet, supplying easily available energy for the microorganisms of the rumen to synthesize microbial protein. According to Abrahamse et al [30], diets with a higher proportion of NFC, even with a similar intake of digestible energy provide a higher supply of fermentable carbohydrates and this can result in a higher level of protein in the milk.

As palatability is defined by the physical and chemical characteristics which “tease appetite”, besides, ruminant select their feed based on flavor and color, these observations prove the necessity of more studies with cactus *Opuntia* to unfold the reasons why the animals do not ingest the same amount of DM than when they are offered the cactus *Nopalea*.

The efficiency of concentrate usage becomes better when diets with 20% of concentrate, 50% of cactus *Opuntia* and 30% of forage sorghum silage (4.91 kg of milk/kg of concentrate) are used when compared to the other levels of concentrate in diets (4.0, 3.5, and 3.07 kg of milk/kg of concentrate). This fact can be explained by the small difference in the MY (Table 7) and the increase in the concentrate intake with the higher levels of concentrate in the diets. Also, for each US$ spent to feeding the cows it will return 0.49, 0.57, 0.53, 0.52, 0.50 US$/d of milk, considering the control diet and, 20%, 24%, 28%, and 32% of concentrate inclusion in *Opuntia* diets, respectively.

Despite the lowest return calculated for 32% concentrate inclusion, it should be considered the daily gain of 70 g promoted by this diet, which implies in better reproductive herd efficiency and important energy source to replace the body reserve at the beginning of the lactation. Increase in the total volume of produced milk per day (1.5 kg/d more than the volume promoted by the 20% concentrate inclusion). In the other hand, a lower concentrate level could be used in the production system to feed dairy cows in late lactation stage.

Thus, *Opuntia* is a viable option in the dairy system and the main advantages are the agronomic characteristics, once it demands fewer nutrients and is more tolerant of hydric stress. Also, *Opuntia* presents a higher production of dry matter per unit area (37 t of DM/ha/2 yr) than *Nopalea* (21 t of DM/ha/2 yr) [6], which may offset the concentrate feeding costs.

In conclusion, to Girolando cows producing 14 kg/d with 3.5% of fat, it is recommended the inclusion of 32% of concentrate in cactus *Opuntia*-based diets to achieve similar performance to those fed cactus *Nopalea*-based diet with 20% of concentrate. In addition, the concentrate inclusion on cactus *Opuntia*-based diets promotes a linear increase in MY.

## IMPLICATIONS

The study provides applicable animal production and nutrition information relating to the new genotype of cactus cladodes for lactating cow’s diets raising in semiarid regions. Although cactus cladodes have been used around the world in arid lands, there is a necessity to study other genotypes viability to inclusion on the herd diet promoting the production sustainability and agricultural diversity. Despite the use of the new genotype requires more concentrate inclusion on diets to guarantee adequate milk production, the agronomic characteristics, as high green matter production could compensate for this challenge.

## Figures and Tables

**Table 1:** Chemical composition of experimental diet feeds

Item (%)	Sorghum silage	*Opuntia*	*Nopalea*	Ground corn	Soybean meal
Dry matter	33.10	9.07	15.60	86.83	87.56
Organic matter	95.87	86.96	93.06	98.63	93.25
Crude protein	6.58	4.38	3.93	9.00	45.21
Non-fiber carbohydrates	28.44	58.55	65.98	76.17	29.48
Neutral detergent fiber	58.21	23.32	21.71	11.54	13.31

**Table 2:** Ingredient proportion and chemical composition of experimental diets

Items	Control	Concentrate levels (%)			

		20	24	28	32
Ingredients					
*Nopalea*	49.66	0.00	0.00	0.00	0.00
*Opuntia*	0.00	49.53	45.71	41.73	37.53
Forage sorghum silage	30.34	30.47	30.29	30.27	30.47
Ground corn	2.25	3.50	7.90	12.30	16.70
Soybean meal	14.75	13.50	13.10	12.70	12.30
Salt	0.50	0.50	0.50	0.50	0.50
Mineral1)	1.50	1.50	1.50	1.50	1.50
Urea/AS2)	1.00	1.00	1.00	1.00	1.00
Chemical composition of the diet (% dry matter)					
Dry matter	23.12	15.14	16.06	17.15	18.45
Organic matter	91.27	88.32	88.79	89.28	89.79
Neutral detergent fiber	30.67	31.49	30.95	30.46	30.05
Non-fiber carbohydrates	47.46	44.31	45.26	46.16	46.99
Crude protein	13.47	13.24	13.28	13.32	13.37
Total digestible nutrients	64.45	59.41	63.56	62.42	62.74

1) Components: dicalcium phosphate, limestone, salt, sulfur flower, zinc sulfate, copper sulfate; manganese sulfate, potassium iodate, and sodium selenite.

2) 9:1 parts of urea to ammonium sulfate (AS).

**Table 3:** Intake and nutrient digestibility of Girolando cows fed cactus cladodes with different levels of concentrate

Items	Control	Concentrate levels (%)				SEM	p-value		
	
		20	24	28	32		L	Q	D
Daily intake (kg/d)									
Dry matter1)	15.4	12.8*	13.5*	13.8*	14.4*	0.28	0.003	0.798	0.000
Organic matter2)	14.1	11.4*	12.0*	12.4*	12.9*	0.24	<0.001	0.902	0.000
Crude protein3)	2.10	1.75*	1.83*	1.87*	1.97*	0.04	0.001	0.850	0.000
Non-fiber carbohydrates4)	7.48	5.70*	6.15*	6.43*	6.88*	0.13	<0.001	0.964	0.000
Neutral detergent fiber	4.53	4.04	4.16	4.19	4.24	0.15	0.171	0.673	0.248
Total digestible nutrients5)	9.90	7.54*	8.62*	8.63*	9.07*	0.22	<0.001	0.156	0.000
Daily intake (g/kg body weight)									
Dry matter6)	30.4	25.2*	26.3*	26.8*	27.9*	0.06	0.003	0.995	0.001
Neutral detergent fiber	8.97	7.99	8.12	8.15	8.20	0.19	0.455	0.817	0.100
Total apparent digestibility (%)									
Dry matter	63.0	55.9*	61.4	60.3	60.5	1.50	0.073	0.092	0.033
Organic matter	64.8	60.3	64.9	63.4	63.5	1.30	0.173	0.099	0.125
Crude protein	66.0	64.6	65.6	63.7	63.9	2.23	0.689	0.855	0.515
Neutral detergent fiber	48.7	52.2	54.9	52.0	50.9	2.99	0.527	0.423	0.443

SEM, standard error mean; L, linear effect; Q, quadratic effect; D, Dunnett effect.

1) ŷ = 10.191+0,1315x.

2) ŷ = 8.844+0.1285x.

3) ŷ = 1.4+0.0175x.

4) ŷ = 3.807+0.0955x.

5) ŷ = 5.475+0.115x.

6) ŷ = 20.96+0.215x.

* Values differ statistically from the control treatment at level of 5% probability (p<0.05).

**Table 4:** Blood parameters of Girolando cows fed cactus cladodes with different levels of concentrate

Items	Control	Concentrate levels (%)				SEM	p-value		
	
		20	24	28	32		L	Q	D
BUN1) (mg/dL)	22.9	22.3	23.2	21.4	23.7	0.85	0.516	0.396	0.356
Urea (mg/dL)	48.9	47.7	49.6	45.7	50.8	1.81	0.516	0.396	0.356
NEFA2) (mmol/L)	0.28	0.60	0.30	0.05	0.14	0.14	0.013	0.172	0.082
BHBA3) (mmol/L)	0.74	0.75	0.72	0.65	0.64	0.04	0.018	0.755	0.116
Glucose (mg/dL)	52.5	50.3	49.3	51.5	49.1	3.91	0.913	0.798	0.898

SEM, standard error mean; L, linear effect; Q, quadratic effect; D, Dunnett effect; BUN, blood urea nitrogen; NEFA, non-esterified fatty acids; BHBA, beta-hydroxybutyrate.

1) ŷ = 19.7824+0.064624x.

2) ŷ = 1.332–0.0408x.

3) ŷ = 0.95–0.01x.

**Table 5:** Urinary volume, urea nitrogen and microbial protein efficiency in Girolando cows fed cactus cladodes with different levels of concentrate

Items	Control	Concentrate levels (%)				SEM	p-value		
	
		20	24	28	32		L	Q	D
Urinary volume (L/d)	29.5	38.5	29.2	34.6	27.9	5.15	0.260	0.800	0.560
Urea urine (mg/dL)	2,442	2,542	2,445	2,288	2,444	88.5	0.260	0.160	0.390
Urea nitrogen in milk (mg/dL)	12.53	13.39	12.08	11.54	12.16	0.69	0.180	0.170	0.420
Microbial protein synthesis									
Microbial nitrogen (g/d)	192	148	159	160	177	23.3	0.390	0.900	0.690
Microbial crude protein (g/d)	1,201	924	996	1,001	1,109	146	0.390	0.900	0.690
EMPS (g CP/kg TDN)	121	123	116	116	122	15.7	0.960	0.840	0.990

SEM, standard error mean; L, linear effect; Q, quadratic effect; D, Dunnett effect; EMPS, efficiency of microbial protein synthesis; CP, crude protein; TDN, total digestible nutrients.

**Table 6:** Nitrogen balance of Girolando cows fed cactus cladodes with different levels of concentrate

Items	Control	Concentrate levels (%)				SEM	p-value		
	
		20	24	28	32		L	Q	D
Total N intake1) (g/d)	337	279*	293*	299*	316*	6.13	0.001	0.850	0.001
Fecal excretion									
Total N (g/d)	115	99.2	99.7	109	113	7.72	0.149	0.810	0.416
Total N (% intake)	33.9	35.5	34.4	36.4	36.2	2.23	0.689	0.855	0.426
Urinary excretion									
Total N (g/d)	29.9	25.2	21.9	19.6	19.9	5.17	0.434	0.725	0.465
Total N (% intake)	8.73	8.68	7.73	6.73	6.45	1.63	0.300	0.838	0.486
Milk excretion									
Total N2) (g/d)	69.6	59.2*	62.7*	65.1	68.0	1.44	0.001	0.862	0.001
Total N (% intake)	20.8	21.3	21.5	21.8	21.7	0.47	0.468	0.746	0.436
Nitrogen balance									
N retained (g/d)	122	95.6	109	106	114	10.91	0.177	0.792	0.463
N retained (% intake)	36.5	34.6	36.4	35.1	35.7	3.27	0.863	0.828	0.479
NE (g N milk/g N intake)	0.21	0.21	0.21	0.22	0.22	0.00	0.468	0.746	0.436

SEM, standard error mean; L, linear effect; Q, quadratic effect; D, Dunnett effect; N, nitrogen; NE, nitrogen efficiency.

1) ŷ = 222.14+2.8747x.

2) ŷ = 45.106+0.7178x.

* Values differ statistically from the control treatment at level of 5% probability (p<0.05).

**Table 7:** Milk yield and composition, and feed efficiency of Girolando cows fed cactus cladodes with different levels of concentrate

Items	Control	Concentrate levels (%)				SEM	p-value		
	
		20	24	28	32		L	Q	D
FCMY (kg/d)	14.0	12.5*	12.8*	13.4*	14.0	0.28	0.003	0.300	0.001
Milk composition (g/100 g)									
Fat	4.18	3.94*	3.76*	3.83*	3.94*	0.07	0.815	0.052	0.001
Protein	3.50	3.25*	3.26*	3.27*	3.32*	0.03	0.099	0.489	0.000
Lactose	4.42	4.43	4.44	4.46	4.47	0.02	0.253	1.000	0.525
Total solids	13.1	12.6*	12.5*	12.6*	12.7*	0.08	0.261	0.052	0.000
Casein	2.75	2.51	2.52	2.55	2.59	10.88	0.624	0.273	0.422
Urea nitrogen (mg/dL)	12.5	13.4	12.1	11.5	12.2	0.69	0.180	0.172	0.425
Efficiency									
Feed efficiency1) (kg FCMY/kg DMI)	0.94	0.97	0.93	0.95	0.97	0.02	0.887	0.197	0.589
Efficiency of concentrate usage2) (kg FCMY/kg concentrate)	4.56	4.91*	4.00*	3.50*	3.07*	0.08	0.001	0.234	0.001

SEM, standard error mean; L, linear effect; Q, quadratic effect; D, Dunnett effect; FCMY, 3.5% fat-corrected milk yield; DMI, dry matter intake.

1) ŷ = 9.782+0.1305x.

2) ŷ = 8.499+0.0085x.

* Values differ statistically from the control treatment at level of 5% probability (p<0.05).
